# Mathematical modelling of a hypoxia-regulated oncolytic virus delivered by tumour-associated macrophages

**DOI:** 10.1016/j.jtbi.2018.10.044

**Published:** 2019-01-14

**Authors:** Michael A. Boemo, Helen M. Byrne

**Affiliations:** a Sir William Dunn School of Pathology, University of Oxford, South Parks Road, Oxford, OX1 3RE, United Kingdom; b Mathematical Institute, University of Oxford, Andrew Wiles Building, Woodstock Road, Oxford, OX2 6GG, United Kingdom

**Keywords:** Mathematical modelling, Theory of mixtures, Oncolytic adenovirus, Tumour-associated macrophages

## Abstract

Tumour hypoxia has long presented a challenge for cancer therapy: Poor vascularisation in hypoxic regions hinders both the delivery of chemotherapeutic agents and the response to radiotherapy, and hypoxic cancer cells that survive treatment can trigger tumour regrowth after treatment has ended. Tumour-associated macrophages are attractive vehicles for drug delivery because they localise in hypoxic areas of the tumour. In this paper, we derive a mathematical model for the infiltration of an in vitro tumour spheroid by macrophages that have been engineered to release an oncolytic adenovirus under hypoxic conditions. We use this model to predict the efficacy of treatment schedules in which radiotherapy and the engineered macrophages are given in combination. Our work suggests that engineered macrophages should be introduced immediately after radiotherapy for maximum treatment efficacy. Our model provides a framework that may guide future experiments to determine how multiple rounds of radiotherapy and macrophage virotherapy should be coordinated to maximise therapeutic responses.

## Highlights

• A continuum model of macrophages releasing an oncolytic virus within a tumour spheroid.
• Predictive modelling of this treatment given in combination with radiotherapy.
• Investigation into how radiotherapy and oncolytic virotherapy should be scheduled.

## 1 Introduction

Surgical removal of a tumour accompanied by adjuvant radiotherapy is a mainstay of cancer treatment, but tumour hypoxia Höckel and Vaupel (2001) limits the efficacy of radiotherapy in a number of ways. Radiotherapy produces oxygen free radicals that can cause DNA damage in tumour cells; a lack of oxygen makes radiation therapy less effective Harrison et al. (2002). In addition, hypoxia modifies the hypoxia-inducible factor 1 (HIF-1) pathway to make the tumour microenvironment antioxidant-rich Sattler and Mueller-Klieser (2009). This helps to negate the DNA damaging effects of the reactive oxygen radicals created by the radiotherapy, leading to radioresistance of the tumour. For these reasons, the HIF-1 pathway is a prime therapeutic target Giaccia et al. (2003); Gillespie et al. (2015); Koyasu et al. (2018); Nakashima et al. (2017); Semenza (2012). In addition to limiting the efficacy of radiotherapy, hypoxia poses a number of other challenges for conventional treatment: poor vascularisation in hypoxic areas inhibits the delivery of chemotherapeutic agents Carmeliet et al. (1998); Minchinton and Tannock (2006), antitumour immune responses are often disrupted Noman et al. (2015), and hypoxic cells that survive treatment can stimulate other tumour cells to regrow Covello et al. (2006); Harris (2002). For these reasons, tumour hypoxia is associated with a poor prognosis for many cancers Birner et al. (2000); Bos et al. (2003); Brizel et al. (1997); Vaupel and Mayer (2007).

In recent years, the effort to improve treatment for hypoxic tumours has led to new radiotherapy techniques Troost et al. (2017) as well as exploiting tumour hypoxia for targeted treatment delivery Brown and Wilson (2004); Shannon et al. (2003). For example, a non-active prodrug can be converted into a cytotoxic free radical when it encounters hypoxic conditions Denny (2000). The prodrug is converted into a free radical by oxidising agents, such as cytrochrome P450s Meunier et al. (2004). In well-oxygenated tissues, the added electron is quickly reduced away by the ambient oxygen molecules. Under hypoxic conditions, however, the oxidised drug remains in its cytotoxic free radical form and can damage tumour cell DNA. An alternative form of therapy uses an oncolytic adenovirus with HIF-dependent replication, restricting viral replication to hypoxic tissue Post and Van Meir (2003). The oncolytic virus then induces cell death pathways in tumour cells and causes an increased immune response within the hypoxic region Stojdl et al. (2000).

While these targeted therapies are promising developments, the poor vascularisation of hypoxic areas still poses a challenge for the effective delivery of both prodrugs and viral therapy. Vehicles for targeted drug delivery are needed and macrophages have been shown to be promising candidates. Tumours release a number of chemoattractants, attracting monocytes that differentiate into tumour-associated macrophages (TAMs) once inside the tumour Richards et al. (2013). The role played by TAMs in tumour progression is controversial and complex, as TAMs have been shown to both promote and inhibit tumour growth in different contexts Lewis and Murdoch (2005). Macrophages with the M1 phenotype can inhibit growth by lysing tumour cells, promoting inflammation, and presenting antigens to T cells. In contrast, macrophages with the M2 phenotype promote tumour growth, in part by promoting angiogenesis and facilitating tissue repair Quatromoni and Eruslanov (2012). TAMs are known to localise in hypoxic regions of the tumour Murdoch, Giannoudis, Lewis, 2004, Murdoch, Muthana, Lewis, 2005 and this has been the subject of theoretical study: Owen et al. have created a phenomenological model of macrophage infiltration into a tumour spheroid Owen et al. (2004). Webb et al. extended this model to include the macrophage delivery of a hypoxia-activated prodrug Webb et al. (2007).

In addition to prodrugs, macrophages can also be used to deliver an oncolytic adenovirus to the hypoxic tumour cells Muthana et al. (2011). In this treatment scheme, the gene E1A, or adenovirus early region 1A, is expressed by an adenovirus to enable viral reproduction: it drives the host cell into S-phase and triggers the expression of other early viral genes Nevins (1981); Radko et al. (2015). When macrophages are cotransduced with E1A/B regulated by hypoxia-associated transcription factors and an E1A-deficient oncolytic virus, the virus replicates only when the macrophages express E1A/B under hypoxic conditions. Adding these “engineered” macrophages as adjuvant therapy following chemotherapy or radiotherapy has been shown to be more effective at abolishing prostate tumours in mice than chemotherapy or radiation alone Muthana, Giannoudis, Scott, 2011, Muthana, Rodrigues, Chem, Welford, Hughes, Tazzyman, Essand, Morrow, E., 2013. Deciding when the engineered macrophages should be delivered in relation to chemotherapy, as well as the dose at which they should be administered, remain important open questions. The mathematical model developed and analysed in this paper provides a methodology for addressing such questions. Suitably parametrised against experimental data, it could be used to compare the efficacy of several different treatment schedules. For example, the model suggests that there may be an optimal time when engineered macrophages should be introduced following primary treatment with radiotherapy.

Our model is specialised to describe a tumour spheroid’s growth and response to treatment. Grown in vitro, tumour spheroids are a useful experimental model system for drug penetration in an avascular tumour Minchinton and Tannock (2006); Sutherland (1988). They are also well-suited for mathematical modelling: Tumour spheroids have a simple geometry and a well-defined structure that consists of a proliferating outer layer of cells that surrounds a necrotic core. For example, Ward and King modelled avascular spheroid growth via a system of coupled nonlinear partial differential equations for live tumour cells, dead cells, and nutrient Ward and King (1997). Sherratt and Chaplain incorporated a new scheme of cell motility by allowing for extracellular space in the tumour Sherratt and Chaplain (2001). Breward et al. added vasculature as an additional phase to model tumour angiogenesis Breward et al. (2002). Byrne and Preziosi derived a model for tumour spheroid growth using the theory of mixtures Byrne (2012); Byrne and Preziosi (2003). Some recent models have been made treatment-specific, such as the reaction-diffusion model for the movement of macrophages within a tumour spheroid Owen et al. (2004) and an extension of this model that uses macrophages as prodrug delivery vehicles Webb et al. (2007). The principles used in these models have also been used in more complex multiscale approaches (see, for example, Jiang et al. (2005)).

Due to its widespread use as a frontline treatment for cancer, there are many mathematical models of radiotherapy. Of these, the linear-quadratic model is the most popular Fowler (1989). There are fewer mathematical models of virotherapy, reflecting the fact that it is a less widely used treatment (see Wodarz, 2016 for a recent review). Our model builds upon and complements existing theoretical models for tumour responses to radiotherapy and oncolytic virotherapy. In particular, Lewin et al. modelled how the spatial distribution of oxygen within a tumour microenvironment affected the efficacy of radiotherapy Lewin et al. (2018). Wu et al. and Jenner et al. modelled the infection of tumour cells by an oncolytic virus in the absence of radiotherapy or macrophages, which led to infected and uninfected subpopulations of tumour cells Jenner et al. (2018); Wu et al. (2001). Crivelli et al. and Wares et al. introduced a cell cycle component into a model of virotherapy with the vesicular somatisis virus (VSV). This particular virus has antitumour efficacy in a range of human cell lines, but the virus is unable to replicate in quiescent *G*_0_ cells Crivelli et al. (2012); Wares et al. (2014). Eftimie et al. modelled the effect that polarisation of tumour-associated macrophages into M1 or M2 had on the effectiveness of oncolytic virus treatment Eftimie and Eftimie (2018).

The remainder of this paper is organised as follows. In Section 2 we use the theory of mixtures to develop a model for the in vitro growth of a tumour spheroid. This development follows the method introduced in Byrne (2012); Byrne and Preziosi (2003); Webb et al. (2007). The model is extended in Section 3 to account for the tumour’s response to treatment with radiotherapy and/or macrophage-delivered virotherapy. In Section 4 we present simulation results for a variety of treatment schedules. We also identify promising treatment schedules that merit further experimental investigation. The paper concludes in Section 5 with a summary of our results and a discussion of the advantages and disadvantages of this modelling approach.

## 2 Model development using the theory of mixtures

### 2.1 Model equations

In this section we derive a model for the in vitro growth of an avascular tumour spheroid that is infiltrated by macrophages. We generally follow the approach used in Byrne (2012); Byrne and Preziosi (2003) by developing a multiphase model of tumour growth using the theory of mixtures; we add an additional phase for macrophages following Webb et al. (2007). The tumour is assumed to comprise three interacting phases: macrophages, tumour cells, and extracellular material. While the model introduced in Webb et al. (2007) assumed that all phases move with a constant advection velocity, we introduce distinct velocities for each phase. We therefore denote by *l, m*, and *n* the volume fractions of the macrophages, tumour cells and extracellular material, respectively. The velocity, stress tensor, and pressure for each phase are denoted by **v**_*i*_, ***σ***_*i*_, *P_i_* for i=l,m,n. The governing equations are derived by applying conservation of mass and momentum to each phase; the model is closed by making constitutive assumptions about the material properties of each phase, interactions between the phases, and the factors regulating cell proliferation and death.

Applying the principle of mass balance to the three constituent phases, *l, m*, and *n*, supplies:

(1) $$\begin{array}{cc} \frac{\partial l}{\partial t} & =S_{l}-\nabla \cdot \left(lv_{l}\right), \end{array}$$

(2) $$\begin{array}{cc} \frac{\partial m}{\partial t} & =S_{m}-\nabla \cdot \left(mv_{m}\right), \end{array}$$

(3) $$\begin{array}{cc} \frac{\partial n}{\partial t} & =S_{n}-\nabla \cdot \left(nv_{n}\right). \end{array}$$

In Eqs. (1)–(3), the functions *S_l_, S_m_*, and *S_n_* represent the net rates of production for each phase. Equations for **v**_*l*_, **v**_*m*_, and **v**_*n*_ are derived by applying conservation of momentum to each phase and neglecting inertial effects:

(4) $$\begin{array}{cc} 0 & =\nabla \cdot \left(l\sigma_{l}\right)+F_{ln}+F_{lm}+P\nabla l+F_{\text{a}}, \end{array}$$

(5) $$\begin{array}{cc} 0 & =\nabla \cdot \left(m\sigma_{m}\right)+F_{mn}+F_{ml}+P\nabla m, \end{array}$$

(6) $$\begin{array}{cc} 0 & =\nabla \cdot \left(n\sigma_{n}\right)+F_{nm}+F_{nl}+P\nabla n. \end{array}$$

In Eqs. (4)–(6), the first term represents the internal forces in each phase while **F**_*ij*_ (i,j=l,m,n;i≠j) denotes the inter-phase force exerted on phase *j* by phase *i*, noting that $F_{ij}=-F_{ji}$. The macrophages are subject to an additional chemoattractive force denoted by **F**_*a*_ which is discussed below. Interfacial effects are modelled by the terms *P*∇*l, P*∇*m*, and *P*∇*n* where *P* is the interfacial pressure: these interfacial contributions arise by taking a continuum limit over discrete cells (see Drew and Segel, 1971 for more details). As is standard in multiphase models of this type, *P* is determined implicitly by assuming that there are no voids in the tumour so that,

(7) l(x,t)+m(x,t)+n(x,t)=1forallx,t.

Two additional phases, oxygen *c* and chemoattractant *a*, are assumed to comprise sufficiently small molecules that they occupy negligible volume and, as such, do not contribute to the no-voids constraint defined by Eq. (7). They are modelled phenomenologically using the following reaction-diffusion equations:

(8) $$\begin{array}{cc} \frac{\partial c}{\partial t} & =D_{c}\nabla^{2}c+S_{c}, \end{array}$$

(9) $$\begin{array}{cc} \frac{\partial a}{\partial t} & =D_{a}\nabla^{2}a+S_{a}. \end{array}$$

The positive parameters *D_c_* and *D_a_* denote the diffusion coefficients (assumed to be constant) of oxygen and the chemoattractant, respectively. As in Eqs. (1)–(3), the terms *S_c_* and *S_a_* represent the net rates of production of oxygen and chemoattractant.

### 2.2 Constitutive assumptions

Eqs. (1)–(9) are closed by imposing suitable boundary and initial conditions (see Section 2.3) and by making constitutive assumptions about the material properties of the volume-occupying phases, the drag forces **F**_*ij*_, and the chemotactic force **F**_*a*_. We begin by specifying functional forms for the net rates of production *S_i_* associated with each phase.

The system is assumed to be closed so that no mass is supplied to, or removed from, the system. Thus, at all points within the tumour,

(10) $$S_{l}+S_{m}+S_{n}=0.$$

When defining *S_l_* and *S_m_*, it is convenient to introduce the “switch” function,

$$\beta \left(A,B\right)=\frac{A^{\alpha }\left(B^{\alpha }+1\right)}{B^{\alpha }+A^{\alpha }}.$$

The “steepness” of this sigmoidal function depends on the parameter *α* > 0, and *B* acts as a “threshold” such that *β*(*A, B*) reaches half of its maximum value when A=B. The rate of tumour cell proliferation $p_{m}=p_{m}\left(m,c\right)$ is defined as

(11) $$p_{m}\left(m,c\right)={\hat{p}}_{m}\beta \left(c,c_{p}\right)\left[1-\beta \left(m,m_{p}\right)\right],$$

where ${\hat{p}}_{m}$ is a positive constant that scales the proliferation rate. In Eq. (11), we assume that the rate of tumour cell proliferation increases with the local oxygen concentration and decreases when the tumour cells are densely packed. Following Webb et al. (2007), the functions *d_l_*(*c*) and *d_m_*(*c*) model the rate at which macrophages and tumor cells die due to lack of oxygen:

(12) $$\begin{array}{cc} d_{l}\left(c\right) & ={\hat{d}}_{l}\left[1-\beta \left(c,c_{c}\right)\right], \end{array}$$

(13) $$\begin{array}{cc} d_{m}\left(c\right) & ={\hat{d}}_{m}\left[1-\beta \left(c,c_{c}\right)\right]+\hat{k}l\left[1-\beta \left(c,c_{p}\right)\right], \end{array}$$

wherein both *c_c_* and *c_p_* are constant oxygen thresholds. Positive constants ${\hat{d}}_{l}$ and ${\hat{d}}_{m}$ scale the death rate of macrophages and tumour cells, respectively. The constant $\hat{k}$ scales the death rate at which macrophages lyse hypoxic tumour cells.

The terms *S_l_* and *S_m_* that appear in Eqs. (1)–(3) represent the net rates of production for macrophages and tumour cells. They are defined by summing the proliferation and death terms:

(14) $$\begin{array}{cc} S_{l} & =-ld_{l}\left(c\right), \end{array}$$

(15) $$\begin{array}{cc} S_{m} & =mp_{m}\left(m,c\right)-md_{m}\left(c\right). \end{array}$$

An expression for *S_n_* follows naturally from Eq. (10) which supplies $S_{n}=-\left(S_{l}+S_{m}\right)$.

The net rates of production for oxygen and the chemoattractant are specified in a similar fashion. The oxygen consumption rate *S_c_* in Eq. (8) is given by

$$S_{c}=-c\left[{\hat{d}}_{cl}l+{\hat{d}}_{cm}m\right]-{\hat{d}}_{cp}mp_{m}\left(m,c\right),$$

where the positive constants ${\hat{d}}_{cl},$ ${\hat{d}}_{cm},$ and ${\hat{d}}_{cp}$ scale the rates at which macrophages and tumour cells consume oxygen. The first term models oxygen consumption by tumour cells and macrophages while the second term models oxygen consumption by cell division. The net rate of production of chemoattractant *S_a_* in Eq. (9) is given by,

$$S_{a}=\left[1-\beta \left(c,c_{p}\right)\right]\left[{\hat{p}}_{al}l+{\hat{p}}_{am}m\right]-\lambda_{a}a,$$

where ${\hat{p}}_{al},$ ${\hat{p}}_{am},$ and *λ_a_* are positive constants. The function *S_a_* accounts for chemoattractant production by macrophages and tumour cells under hypoxic conditions. It also accounts for the natural breakdown of chemoattractant at rate *λ_a_*.

Following Breward, Byrne, Lewis, 2001, Breward, Byrne, Lewis, 2003 we assume that the interfacial/drag forces in Eqs. (4)–(6) depend linearly on the relevant inter-phase velocities so that:

(16) $$\begin{array}{cc} F_{mn} & =kmn(v_{n}-v_{m}), \end{array}$$

(17) $$\begin{array}{cc} F_{ln} & =kln(v_{n}-v_{l}), \end{array}$$

(18) $$\begin{array}{cc} F_{lm} & =kml(v_{m}-v_{l}). \end{array}$$

For simplicity, the constant of proportionality *k* in Eqs. (16)–(18) is assumed to be the same for each force. The chemoattractive force **F**_*a*_ drives macrophages up spatial gradients in the chemoattractant distribution and is assumed to be proportional to the volume fraction of macrophages *l* and spatial gradient of the chemoattractant concentration so that

$$F_{a}=\chi l\nabla a,$$

wherein the parameter *χ* > 0 governs the strength of chemoattraction.

Before specifying the stress tensors ***σ***_*i*_ (for i=l,m,n), we note that the above equations are written in terms of an arbitrary, three-dimensional coordinate system. For simplicity, we restrict attention to one-dimensional, radially symmetric spherical coordinates and associate the spatial position with a single, independent variable *r* that specifies distance from the centre of the spherically symmetric tumour. Therefore, all phases are defined at points (*r, t*) instead of (**x**, *t*).

Building on established work (see, for example, Byrne, 2012) we view all volume-occupying phases as inviscid fluids and associate with each of them an interfacial pressure denoted by *P_l_, P_m_*, and *P_n_*. For simplicity, fix $P_{n}=P$ so that the pressure in the extracellular material is identical to that in the fluid surrounding the tumour. As in Breward, Byrne, Lewis, 2001, Breward, Byrne, Lewis, 2003, we assume that each cellular phase is similar in form to the extracellular material save for additional correction terms *D_l_* and *D_m_* that characterise the way in which the macrophages and tumour cells differ from inert bags of fluid. In what follows, we will show that *D_l_* and *D_m_* can be interpreted as diffusion coefficients for the two cell species (see Eqs. (32)–(33) below). In one-dimensional radially symmetric coordinates, the stress tensors reduce to scalars given by,

(19) $$\begin{array}{cccc} \sigma_{n} & =-P_{n} & & =-P, \end{array}$$

(20) $$\begin{array}{cccc} \sigma_{l} & =-P_{l} & & =-(P+D_{l}), \end{array}$$

(21) $$\begin{array}{cccc} \sigma_{m} & =-P_{m} & & =-(P+D_{m}). \end{array}$$

### 2.3 Boundary and initial conditions

As the tumour changes in size, the domain on which the model equations are solved also changes: The model is a moving boundary problem where r=R(t) denotes the spatial position of the outer tumour radius (i.e., where tumour cells meet the surrounding culture medium). We assume that this boundary moves with the same velocity as the tumour cell phase so that:

(22) $$\frac{dR}{dt}=v_{m}\left(R,t\right).$$

Following Webb et al. (2007) we impose mixed boundary conditions for the macrophages, extracellular material, and chemoattractant on the moving boundary r=R(t). We assume that the flux of each phase across the moving boundary is diffusive, being proportional to the difference between the phase’s concentration on the boundary and the concentration of the phase in the culture medium surrounding the tumour (assumed constant). We further assume that tumour boundary is highly permeable to oxygen and therefore impose a Dirichlet boundary condition, fixing the oxygen concentration. Combining these assumptions gives

(23) $$\begin{array}{cc} -l(v_{l}-v_{m}) & =h_{l}\left(l_{\infty }-l\right)\\ -n(v_{n}-v_{m}) & =h_{n}\left(n_{\infty }-n\right)\\ c & =c_{\infty }\\ \frac{\partial a}{\partial r} & =h_{a}\left(a_{\infty }-a\right) \end{array}\}\;\text{on}\;r=R(t).$$

The constants *h_l_, h_n_*, and *h_a_* represent the permeability of the tumour boundary to macrophages, extracellular material, and chemoattractant, respectively. The constants *l*_∞_, *n*_∞_, *c*_∞_, and *a*_∞_ represent the phase concentrations in the culture medium surrounding the tumour. We assume further that the tumour is symmetric about its centre (r=0) and, accordingly, impose the following boundary conditions there:

(24) $$v_{l}=v_{m}=\frac{\partial c}{\partial r}=\frac{\partial a}{\partial r}=0\;\text{on}\;r=0.$$

We close the governing equations by prescribing the initial distributions of tumour cells, macrophages, oxygen, and chemoattractant for all 0 ≤ *r* ≤ *R*(0) wherein the initial radius *R*(0) is also prescribed. Thus, we have

(25) $$m\left(r,0\right)=m_{0},\;l\left(r,0\right)=0,\;c\left(r,0\right)=c_{\infty },\;a\left(r,0\right)=0,\;R\left(0\right)=R_{0}.$$

In Eq. (25), we have assumed that the tumour cell volume fraction is spatially uniform for 0 ≤ *r* ≤ *R*(0) and that the tumour is initially devoid of macrophages and chemoattractant. We assume further that the initial tumour radius *R*_0_ is sufficiently small that the tumour is well-oxygenated with no hypoxic regions; oxygen has a spatially-uniform distribution that is consistent with the oxygen tension in the surrounding culture medium where $c=c_{\infty }$.

### 2.4 Model simplification

We reduce our model a system of diffusion-advection equations by using the momentum balances in Eqs. (4)–(6) to derive expressions for the phase velocities *v_l_, v_m_*, and *v_n_* that appear in the mass balance Eqs. (1) – (3). Summing the mass balance equations and using the no-voids assumption gives

(26) $$v_{l}l+v_{m}m+v_{n}n=0,$$

where $v_{l}=v_{m}=v_{n}=0$ at r=0 by symmetry. Substituting for *σ_m_* in Eq. (5), the momentum balance equation for *m* supplies the following expression for *v_m_*:

(27) $$v_{m}=-\frac{1}{k}\left(\frac{\partial P}{\partial r}+\frac{D_{m}}{m}\frac{\partial m}{\partial r}\right).$$

A similar derivation supplies the following expression for *v_l_*:

(28) $$v_{l}=-\frac{1}{k}\left(\frac{\partial P}{\partial r}+\frac{D_{l}}{l}\frac{\partial l}{\partial r}-\chi \frac{\partial a}{\partial r}\right).$$

Summing the momentum balance Eqs. (4)–(6) shows that

(29) $$\frac{\partial P}{\partial r}=\chi l\frac{\partial a}{\partial r}-D_{l}\frac{\partial l}{\partial r}-D_{m}\frac{\partial m}{\partial r}.$$

Using this equation to eliminate *P* from the expressions for *v_m_* and *v_l_* provides

(30) $$\begin{array}{cc} v_{l} & =\frac{1}{k}\left(D_{l}\left(1-\frac{1}{l}\right)\frac{\partial l}{\partial r}+D_{m}\frac{\partial m}{\partial r}+\left(1-l\right)\chi \frac{\partial a}{\partial r}\right), \end{array}$$

(31) $$\begin{array}{cc} v_{m} & =\frac{1}{k}\left(D_{l}\frac{\partial l}{\partial r}+D_{m}\left(1-\frac{1}{m}\right)\frac{\partial m}{\partial r}-\chi l\frac{\partial a}{\partial r}\right). \end{array}$$

We use Eqs. (30)–(31) to substitute *v_l_* and *v_m_* in the mass balance Eqs. (1)–(3), which results in the following diffusion-advection equations that govern the evolution of the macrophage and tumour cell volume fractions:

(32) $$\begin{array}{cc} \frac{\partial l}{\partial t} & =\frac{1}{r^{2}}\frac{\partial }{\partial r}\left[\frac{r^{2}}{k}\left(D_{l}\left(1-l\right)\frac{\partial l}{\partial r}-lD_{m}\frac{\partial m}{\partial r}-\chi l\left(1-l\right)\frac{\partial a}{\partial r}\right)\right]-S_{l}, \end{array}$$

(33) $$\begin{array}{cc} \frac{\partial m}{\partial t} & =\frac{1}{r^{2}}\frac{\partial }{\partial r}\left[\frac{r^{2}}{k}\left(D_{m}\left(1-m\right)\frac{\partial m}{\partial r}-mD_{l}\frac{\partial l}{\partial r}+\chi lm\frac{\partial a}{\partial r}\right)\right]+S_{m}. \end{array}$$

### 2.5 Model solution

In order to construct numerical solutions of the governing equations, it is convenient first to introduce a coordinate transformation $\left(r,t\right)\to \left(\frac{r}{R(t)},t\right)$ which maps the moving boundary problem onto a fixed domain (see Ward and King, 1997 for more details). The resulting equations are then solved using the method of lines: we use finite difference approximations to discretise spatial derivatives into 100 grid points and obtain a system of time-dependent ordinary differential equations which we solve using a variable-step, variable-order solver; the code is available on request.

The model is comprised of Eqs. (32) - (33) for the movement of macrophages and tumour cells, as well as Eqs. (8) - (9) for the movement of oxygen and chemoattractant. The boundary conditions are given by Eqs. (22) - (23), and suitable initial conditions are given by Eq. (25). We solved these equations using the dimensionless parameter values listed in Table A.1 so that the model solutions exhibited behaviours similar to those reported for the similar models presented in Owen et al. (2004); Webb et al. (2007). In particular, the rate of tumour growth should slow over time, the tumour should develop a necrotic core once it grows large enough that oxygen can no longer penetrate into its centre, and macrophages should localise to this necrotic core. To ensure that the model reproduced the underlying biology, we compared our model solution with the results of experiments from Leek Leek (1999) which quantified in vitro infiltration of macrophages into a tumour spheroid grown from a mouse hepatocellular carcinoma (HEPA-1) cell line. This dataset included time course measurements for the radius of *in vitro* tumour spheroids grown in the absence of macrophages and the time and radius at which these spheroids developed a necrotic core. Leek also performed a separate experiment where fluorescently labelled macrophages were co-culatured with tumour spheroids and the spatial distribution of macrophages within the tumour was measured at a fixed time point.

**Fig. 1:**
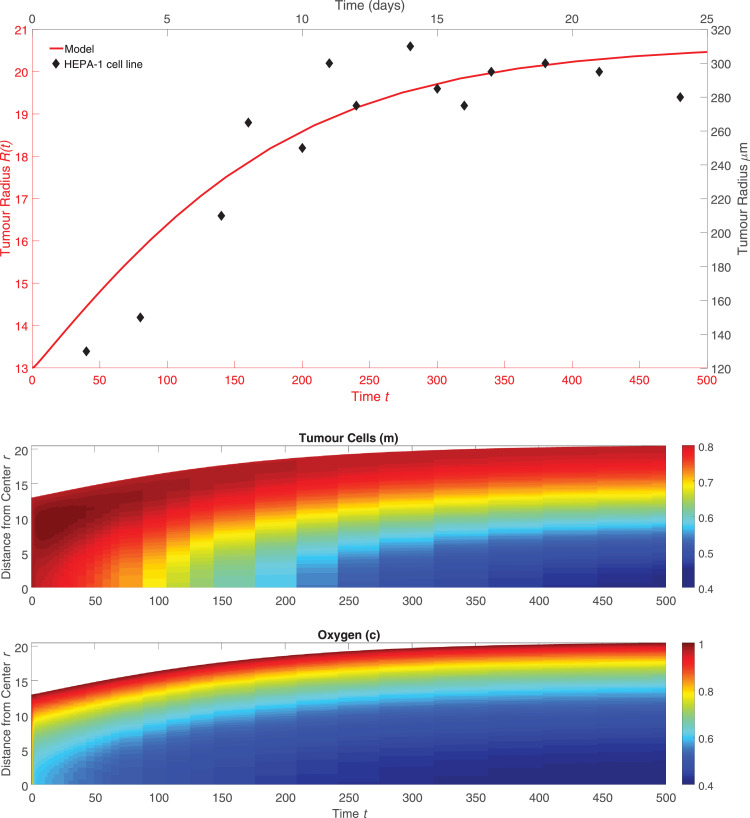
*(upper)* Radius measurements over time of HEPA-1 tumour spheroids where each measurement shown is the average radius of 20 spheroids grown in the absence of macrophages (black, top and right axes) and dimensionless *R*(*t*) curve when the model defined by Eqs. (1) – (3) and (8) – (9) is solved using the parameters in Appendix A with $l_{\infty }=0$ (red, bottom and left axes). Leek computed the standard error of the mean of each radius measurement, but notes that these were negligible on the *μm* scale. *(lower)* The spatial distribution of the tumour cells and oxygen concentration in the model solution associated with the plot of the tumour radius *R*(*t*) shown above. A necrotic core develops at approximately t=100, which is consistent with the observations in Leek (1999). (For interpretation of the references to colour in this figure legend, the reader is referred to the web version of this article.)

In Fig. 1 (upper), we present the model solution for tumour spheroid growth without macrophages which is obtained by setting $l_{\infty }=0$ in Eq. (23). The model solution is compared with the data from Leek (1999) which measured tumour spheroid growth in the absence of macrophages. Fig. 1 (lower) shows the simulated spatial distribution of the tumour cells and oxygen concentration that corresponds to the simulated growth curve in Fig. 1 (upper). In the model solution, a necrotic core develops due to lack of oxygen once *R*(*t*) ≈ 17, which corresponds to approximately 200 *μm*; this is consistent with the experimental data from Leek (1999). We note that Leek observed the partial detachment of cells from the spheroid into the surrounding culture medium at later times, a phenomenon referred to as “disintegration”. For simplicity, our model ignores this effect, but we solve the model on the restricted time domain 0 ≤ *t* ≤ 500 to reflect the limited lifespan of the spheroids and we refer interested readers to Ward and King (1999) for an example of how cell shedding can be incorporated into our model.

**Fig. 2:**
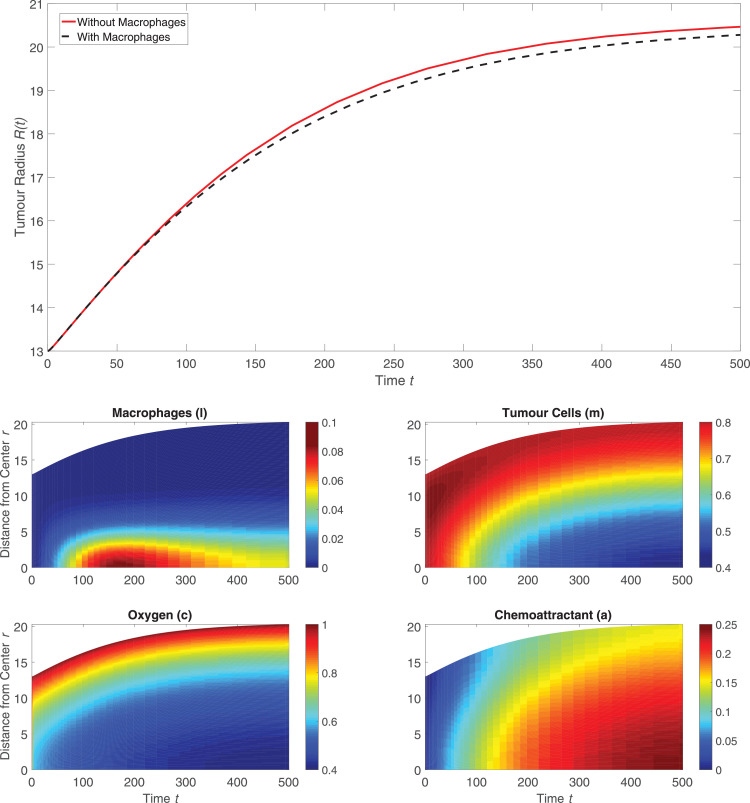
*(upper)* Comparison between *R*(*t*) curves when the model is solved with macrophages (setting $l_{\infty }=0.2,$ black dotted curve) and without macrophages (setting $l_{\infty }=0,$ red solid curve). *(lower)* Series of panels corresponding to the black curve above that show how the spatial distributions of the macrophages, tumour cells, oxygen, and chemoattractant change over time when the model is solved using the default parameter values (see Table A.1). We note that macrophages migrate slowly into the tumour and that, throughout the simulation, the concentration of macrophages remains low but still nonzero in the dark blue region between the tumour’s core and its outer boundary. (For interpretation of the references to colour in this figure legend, the reader is referred to the web version of this article.)

**Fig. 3:**
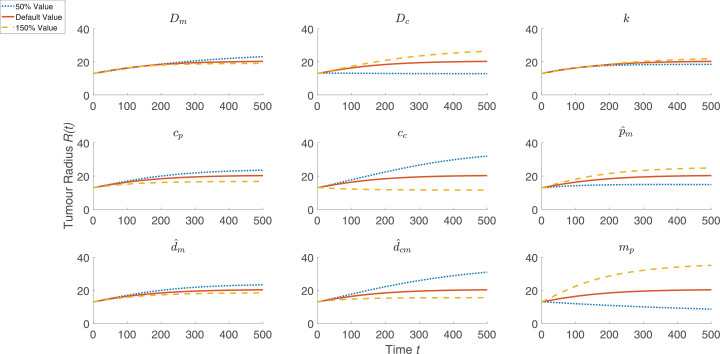
Summary of a one-at-a-time parameter sensitivity analysis of the model defined by Eqs. (1) – (3) and (8) – (9). The nine parameters to which the tumour growth is most sensitive are shown, while results for variation of all parameters are presented in Appendix A (see Fig. A.1). Using the default parameter values specified in Appendix A, and for each model parameter, the governing equations were solved using 50% of that parameter’s default value (blue curve, dotted), the default value (red curve), and 150% of the default value (yellow curve, dashed). (For interpretation of the references to colour in this figure legend, the reader is referred to the web version of this article.)

**Fig. A1:**
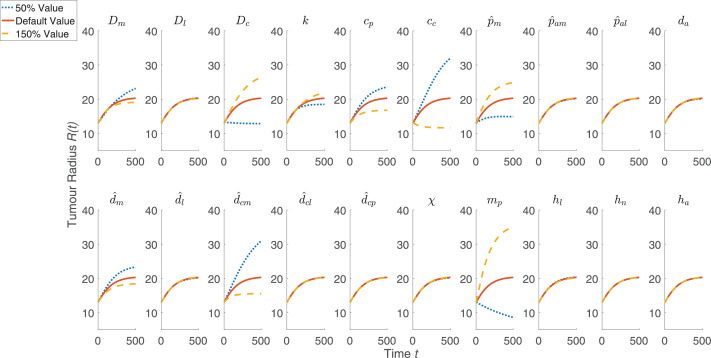
The full results of the one-at-a-time parameter sensitivity analysis to complement the summary in Fig. 3. Using the default parameter values specified in Appendix A, and for each model parameter, the governing equations were solved using 50% of that parameter’s default value (blue curve, dotted), the default value (red curve), and 150% of the default value (yellow curve, dashed) while holding all other parameters fixed at their respective default values. Solutions were numerically stable in all cases. We note that in some cases, the results are sufficiently similar that the three curves are difficult to distinguish. (For interpretation of the references to colour in this figure legend, the reader is referred to the web version of this article.)

To simulate macrophage infiltration into the HEPA-1 tumour spheroids, the model was solved using the default parameter values listed in Appendix A and with *l*_∞_ increased from $l_{\infty }=0$ to $l_{\infty }=0.2$ to account for the presence of macrophages in the surrounding culture medium. Including macrophages has little effect on the tumour size over time, as shown in Fig. 2 (upper). When Leek co-cultured fluorescently labelled mouse macrophages with HEPA-1 tumour spheroids, the macrophages localised near the centre of the tumour and their spatial distribution became more diffuse over time. The spatial distribution of the macrophages in Fig. 2 reveals a similar distribution, whereby for 0 < *t* < 50 the macrophages slowly migrate at low levels to the centre of the spheroid in response to the spatial gradient in chemoattractant which is produced by tumour cells under hypoxia. By t=50, there is notable accumulation of macrophages at the centre of the tumour. To investigate the sensitivity of this model solution to the values of the parameters, we performed a one-at-a-time parameter sensitivity analysis. For each model parameter, the model was solved using that parameter’s default value as stated in Appendix A, 50% of its default value, and 150% of its default value while holding all other parameters fixed at their respective default values. Fig. 3 shows how these parameter changes affected the evolution of the tumour radius for the nine most sensitive parameters (the results for all parameters are shown in Fig. A.1). The parameter changes that most affected tumour growth are those that control tumour cell proliferation (${\hat{p}}_{m},$ *m_p_*), oxygen thresholds (*c_p_, c_c_*), the diffusion coefficient of oxygen (*D_c_*), and the rate of consumption of oxygen by tumour cells (${\hat{d}}_{cm}$). This suggests that the way in which the tumour cells proliferate and interact with oxygen are of primary importance in reproducing growth dynamics that are similar to the experimental results. We note also that our one-at-a-time parameter sensitivity analysis, while advantageous in its simplicity, offers limited insight into the impact that changes in multiple parameters have on the system’s dynamics. It provides qualitative information about those parameters to which the system dynamics are most sensitive; these parameters should be fit when appropriate data becomes available. Such studies and improvements are postponed for future work.

## 3 Macrophage delivery of oncolytic virus

Having established that our mathematical model produces results which are qualitatively similar to experimental data for macrophage infiltration into an avascular tumour spheroid, we now extend it to account for treatment with radiotherapy and macrophage-delivered oncolytic virotherapy.

In the previous section, we assumed that chemoattractant was produced by macrophages and tumour cells under hypoxic conditions. We now assume that radiotherapy, in addition to causing tumour cell death, increases the rate at which the tumour cells secrete chemoattractant; the additional chemoattractant source term can be viewed as a bi-product of the degradation fo the dead cells produced during radiotherapy. Macrophages engineered with an oncolytic virus migrate to regions with high levels of chemoattractant and the virus is released when these macrophages encounter hypoxic regions in the tumour. Free virus can then infect and subsequently kill tumour cells.

To incorporate this behaviour into the model, we partition the tumour cell volume fraction *m* into uninfected tumour cells *m_U_* and infected tumour cells *m_I_*. The no-voids assumption in Eq. (7) generalises to give,

$$m_{U}\left(r,t\right)+m_{I}\left(r,t\right)+l\left(r,t\right)+n\left(r,t\right)=1\;\text{for}\;\text{all}\;r,t.$$

For simplicity, we assume that properties of the infected and uninfected tumour cells, such as their interfacial pressure, are the same.

Radiotherapy is administered at time *t_s_* > 0 before the macrophages are introduced to the tumour culture media, so all tumour cells are uninfected when radiotherapy is administered. The standard linear-quadratic approach Fowler (1989) for modelling radiotherapy gives the fraction of surviving tumour cells after treatment and assumes instantaneous cell death; it is not easily incorporated into our continuum model. Instead, we assume that the death rate of uninfected tumour cells caused by radiation is an exponentially decaying function of time with half-life $r_{t}^{-1}$ which is written as follows:

(34) $$R\left(m,t_{s}\right)=\nu mH\left(t-t_{s}\right)e^{-r_{t}\left(t-t_{s}\right)}.$$

In Eq. (34), *H* denotes the Heaviside step function where H(x)=1 if *x* > 0 and H(x)=0 otherwise. The strength of the radiation dose can be adjusted by varying the scalar parameter *ν*.

The increase in chemoattractant production caused by radiation draws macrophages into the tumour. Under hypoxic conditions, the engineered macrophages release an oncolytic virus with concentration denoted by *ϕ*. We assume further that the virus occupies negligible volume so that its spatial distribution over time is governed by the following reaction-diffusion equation:

(35) $$\frac{\partial \phi }{\partial t}=\frac{1}{r^{2}}\frac{\partial }{\partial r}\left(D_{\phi }\frac{\partial \phi }{\partial r}\right)+S_{\phi }.$$

In Eq. (35) the term *S_ϕ_* represents the net rate of virus production where

(36) $$\begin{array}{ccc} S_{\phi } & = & l{\hat{p}}_{\phi }\left[1-\beta \left(c,c_{c}\right)\right]\\ & & +\eta \left[d_{m}\left(c\right)m_{I}+lm_{I}k\left(c\right)+k_{\phi }m_{I}\right]-\phi \left(1-\beta \left(m_{I}+m_{U},c_{\phi }\right)\right). \end{array}$$

The two production terms in Eq. (36) account for the release of oncolytic virus by macrophages under hypoxia and its release when infected tumour cells die. The positive parameter ${\hat{p}}_{\phi }$ governs the release rate of virus from engineered macrophages under hypoxic conditions, and the constant *η* scales the amount of virus that is released by the death of infected tumour cells. We assume that the virus is short-lived in extracellular material and must be internalised in cells to stay alive; the last term represents the rate at which free virus degrades in the absence of tumour cells.

Uninfected tumour cells can become infected on contact with the oncolytic virus. By analogy with Eq. (33), the volume fraction of uninfected tumour cells is governed by the equation

(37) $$\begin{array}{ccc} \frac{\partial m_{U}}{\partial t} & = & \frac{1}{r^{2}}\frac{\partial }{\partial r}\left[\frac{r^{2}}{k}\left(D_{m}\left(\frac{\partial m_{U}}{\partial r}-m_{U}\left(\frac{\partial m_{U}}{\partial r}+\frac{\partial m_{I}}{\partial r}\right)\right)-m_{U}D_{l}\frac{\partial l}{\partial r}+\chi lm_{U}\frac{\partial a}{\partial r}\right)\right]\\ & & +S_{m_{U}}. \end{array}$$

The net rate of production for uninfected tumour cells $S_{m_{U}}$ is given by

(38) $$S_{m_{U}}=m_{U}\left[p_{m}\left(m_{U}+m_{I},c\right)-d_{m}\left(c\right)-lk\left(c\right)-r_{\phi }\phi \right]-R\left(m_{U},t_{s}\right).$$

This net production rate accounts for cell division in the presence of oxygen, cell death due to low levels of oxygen, lysis by macrophages, and radiotherapy. The term *r_ϕ_ϕ* determines the rate at which uninfected tumour cells become infected by free virus.

In a similar manner, the evolution of the volume fraction of infected tumour cells is modelled as follows:

(39) $$\begin{array}{ccc} \frac{\partial m_{I}}{\partial t} & = & \frac{1}{r^{2}}\frac{\partial }{\partial r}\left[\;\frac{r^{2}}{k}\left(\;D_{m}\left(\;\frac{\partial m_{I}}{\partial r}-m_{I}\left(\;\frac{\partial m_{U}}{\partial r}+\frac{\partial m_{I}}{\partial r}\;\right)\;\right)-m_{I}D_{l}\frac{\partial l}{\partial r}+\chi lm_{I}\frac{\partial a}{\partial r}\;\right)\;\right]\\ & & +S_{m_{I}}, \end{array}$$

wherein the net rate of production of infected tumour cells $S_{m_{I}}$ is defined to be

(40) $$S_{m_{I}}=r_{\phi }\phi m_{U}-m_{I}\left[d_{m}\left(c\right)+lk\left(c\right)+k_{\phi }\right].$$

The leftmost term specifies the rate at which uninfected tumour cells become infected by the oncolytic virus while the positive constant *k_ϕ_* specifies the rate at which infected tumour cells die from viral infection. Like uninfected tumour cells, infected tumour cells can die from a lack of oxygen or lysis by macrophages.

On the tumour boundary, we define the velocity of tumour cells *v*_m*_ to be the weighted average of the velocities of the infected and uninfected tumour cell subpopulations so that,

$$v_{m*}=\frac{m_{I}v_{m_{I}}+m_{U}v_{m_{U}}}{m_{I}+m_{U}}.$$

We assume that this new velocity defines the rate at which the tumour boundary moves so that

$$\begin{array}{ccc} \frac{dR}{dt} & = & v_{m*}\left(R,t\right)\\ & = & \frac{1}{k}{\left(D_{l}\frac{\partial l}{\partial r}+D_{m}\left(1-\frac{1}{m_{U}+m_{I}}\right)\left(\frac{\partial m_{U}}{\partial r}+\frac{\partial m_{I}}{\partial r}\right)-\chi l\frac{\partial a}{\partial r}\right)}_{r=R.} \end{array}$$

For our extended model, Eqs. (24) – (23) are superceded by the following boundary conditions:

(41) $$v_{l}=v_{m_{I}}=v_{m_{U}}=\frac{\partial c}{\partial r}=\frac{\partial a}{\partial r}=\frac{\partial \phi }{\partial r}=0\;\text{on}\;r=0,$$

(42) $$\begin{array}{cc} -l(v_{l}-v_{m*}) & =h_{l}\left(l_{\infty }-l\right)\\ -n(v_{n}-v_{m*}) & =h_{n}\left(n_{\infty }-n\right)\\ v_{m_{I}} & =v_{m_{U}}\\ c & =c_{\infty }\\ \frac{\partial a}{\partial r} & =h_{a}\left(a_{\infty }-a\right)\\ \frac{\partial \phi }{\partial r} & =h_{\phi }\left(\phi_{\infty }-\phi \right) \end{array}\}\;\text{on}\;r=R(t).$$

In Eq. (42), the constant *h_ϕ_* represents the permeability of the tumour boundary to the virus and and the constant *ϕ*_∞_ is the virus concentration in the extracellular fluid surrounding the tumour. Since we assume that the virus can only enter the system via macrophages or by replication inside tumour cells, we fix $\phi_{\infty }=0$. In summary, our model for the response of avascular tumours to treatment with radiotherapy and oncolytic virotherapy comprises the following system of equations:

(43) $$\begin{array}{ccc} \frac{\partial l}{\partial t} & = & \frac{1}{r^{2}}\frac{\partial }{\partial r}\left[\frac{r^{2}}{k}\left(D_{l}\left(1-l\right)\frac{\partial l}{\partial r}-lD_{m}\left(\frac{\partial m_{U}}{\partial r}+\frac{\partial m_{I}}{\partial r}\right)-\chi l\left(1-l\right)\frac{\partial a}{\partial r}\right)\right]\\ & & -ld_{l}\left(c\right), \end{array}$$

(44) $$\begin{array}{cc} \frac{\partial m_{I}}{\partial t}= & \frac{1}{r^{2}}\frac{\partial }{\partial r}\left[\frac{r^{2}}{k}\left(D_{m}\left(\frac{\partial m_{I}}{\partial r}-m_{I}\left(\frac{\partial m_{U}}{\partial r}+\frac{\partial m_{I}}{\partial r}\right)\right)-m_{I}D_{l}\frac{\partial l}{\partial r}+\chi lm_{I}\frac{\partial a}{\partial r}\right)\right] \end{array}$$

(45) $$\begin{array}{cc} & +r_{\phi }\phi m_{U}-m_{I}\left[d_{m}\left(c\right)+lk\left(c\right)+k_{\phi }\right],\\ \frac{\partial m_{U}}{\partial t}= & \frac{1}{r^{2}}\frac{\partial }{\partial r}\left[\frac{r^{2}}{k}\left(D_{m}\left(\frac{\partial m_{U}}{\partial r}-m_{U}\left(\frac{\partial m_{U}}{\partial r}+\frac{\partial m_{I}}{\partial r}\right)\right)-m_{U}D_{l}\frac{\partial l}{\partial r}+\chi lm_{U}\frac{\partial a}{\partial r}\right)\right] \end{array}$$

(46) $$\begin{array}{cc} & +m_{U}\left[p_{m}\left(m_{U}+m_{I},c\right)-d_{m}\left(c\right)-lk\left(c\right)-r_{\phi }\phi \right]-R\left(m_{U},t_{s}\right),\\ \frac{\partial c}{\partial t}= & \frac{1}{r^{2}}\frac{\partial }{\partial r}\left(r^{2}D_{c}\frac{\partial c}{\partial r}\right)-{\hat{d}}_{cl}cl-{\hat{d}}_{cm}c\left(m_{U}+m_{I}\right) \end{array}$$

(47) $$\begin{array}{cc} & -{\hat{d}}_{cp}\left(m_{U}+m_{I}\right)p_{m}\left(m_{U}+m_{I},c\right),\\ \frac{\partial a}{\partial t}= & \frac{1}{r^{2}}\frac{\partial }{\partial r}\left(r^{2}D_{a}\frac{\partial a}{\partial r}\right)+\gamma R\left(m_{U}\right) \end{array}$$

(48) $$\begin{array}{cc} & +\left[1-\beta \left(c,c_{p}\right)\right]\left[{\hat{p}}_{al}l+{\hat{p}}_{am}\left(m_{U}+m_{I}\right)\right]-\lambda_{a}a,\\ \frac{\partial \phi }{\partial t}= & \frac{1}{r^{2}}\frac{\partial }{\partial r}\left(r^{2}D_{\phi }\frac{\partial \phi }{\partial r}\right)+l{\hat{p}}_{\phi }\left[1-\beta \left(c,c_{c}\right)\right] \end{array}$$

(49) $$\begin{array}{cc} & +\eta \left[d_{m}\left(c\right)m_{I}+lm_{I}k\left(c\right)+k_{\phi }m_{I}\right]-\phi \left(1-\beta \left(m_{I}+m_{U},c_{\phi }\right)\right). \end{array}$$

The above equations are solved on a growing domain 0 ≤ *r* ≤ *R*(*t*) where the position of the outer tumour radius *R*(*t*) satisfies

(50) $$\frac{dR}{dt}=\frac{1}{k}{\left(D_{l}\frac{\partial l}{\partial r}+D_{m}\left(1-\frac{1}{m_{U}+m_{I}}\right)\left(\frac{\partial m_{U}}{\partial r}+\frac{\partial m_{I}}{\partial r}\right)-\chi l\frac{\partial a}{\partial r}\;\right)}_{r=R}.$$

Eqs. (43) – (50) are closed by imposing the following initial conditions:

$$\begin{array}{ccc} & & l\left(r,0\right)=0,\;\;m_{I}\left(r,0\right)=0,\;\;m_{U}\left(r,0\right)=0.8,\;\;c\left(r,0\right)=1,\\ & & \;a(r,0)=0,\phi (r,0)=0. \end{array}$$

We note that while initially no macrophages are present in the system, they are introduced at a later point after radiotherapy has been administered.

## 4 Results

**Fig. 4:**
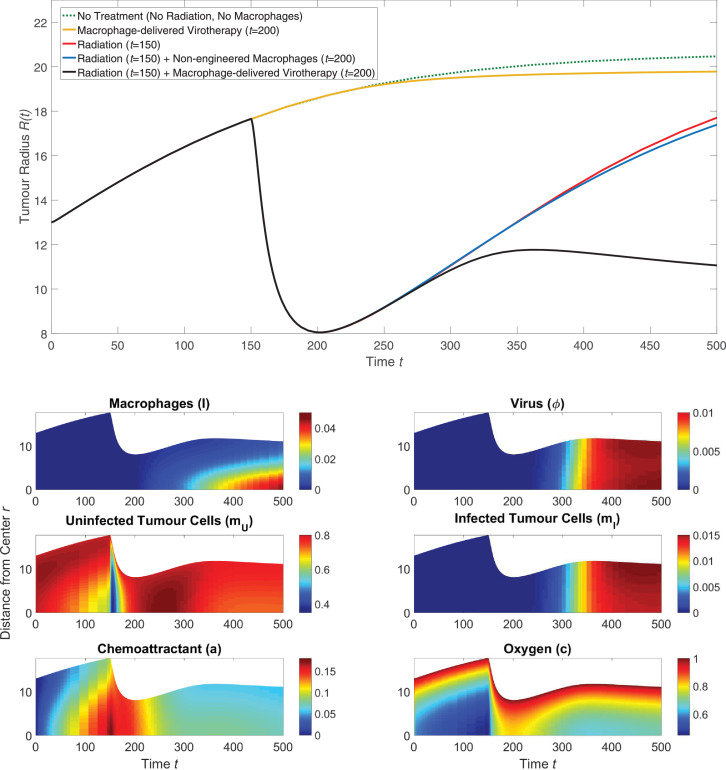
The solution of Eqs. (43) – (50) when solved using the parameter values from Tables A.1 - A.2. *(upper)* Series of plots showing how the tumour radius evolves over time for different treatment schemes. For treatments that used radiation, the radiation was administered at t=150; for treatment that used macrophages, the macrophages were introduced outside the tumour at t=200 by setting $l_{\infty }=0.2$. *(lower)* Series of plots corresponding to the black curve above that show how the spatial distribution of the model phases change over time following radiotherapy delivered at t=150 and macrophage-delivered virotherapy at t=200.

We solved Eqs. (43) – (50) using the parameters in Tables A.1 - A.2 and typical results are presented in Fig. 4. These results suggest that treatment where radiotherapy and macrophage-delivered virotherapy are given together is more successful at reducing tumour size than treatment with only radiotherapy or treatment with only macrophage-delivered virotherapy. Combined radiotherapy and macrophage-delivered virotherapy is also more effective at reducing tumour size than radiotherapy and non-engineered macrophages given together, which suggests that the sustained cell death over time due to the oncolytic virus is a key factor in preventing regrowth of the tumour. By t=500, the size of the tumour given radiotherapy and macrophage-delivered virotherapy is smaller than its starting size at t=0.

The model solution in Fig. 4 suggests the following mechanistic explanation for the effectiveness of radiotherapy and macrophage-delivered virotherapy when given together: We observe an initial decrease in *m_U_*, the uninfected tumour cells, caused by the radiotherapy which reduces the tumour size as the surviving cells concentrate into a smaller tumour mass. This reduction in tumour size allows oxygen to better penetrate into the tumour’s centre, and the cell death from radiotherapy causes an increase in chemoattractant. When macrophages are introduced into the culture medium surrounding the tumour at t=200, they are attracted to the tumour’s centre by the increase in chemoattractant which accompanies tumour cell death from radiotherapy. The rate of macrophage infiltration is further increased by the reduction in tumour radius, as the macrophages have less distance to travel to reach the centre. Oxygen levels at the tumour’s centre fall again as the tumour starts to regrow. This, in turn, further attracts engineered macrophages and stimulates them to release an oncolytic virus that infects and kills tumour cells as it diffuses through the tumour mass. While radiotherapy kills a higher number of tumour cells over a short timescale, virotherapy causes sustained tumour cell death over a long timescale; this prevents regrowth of the tumour. The sustained cell death from virotherapy causes the tumour size to decrease again by t=500.

**Fig. 5:**
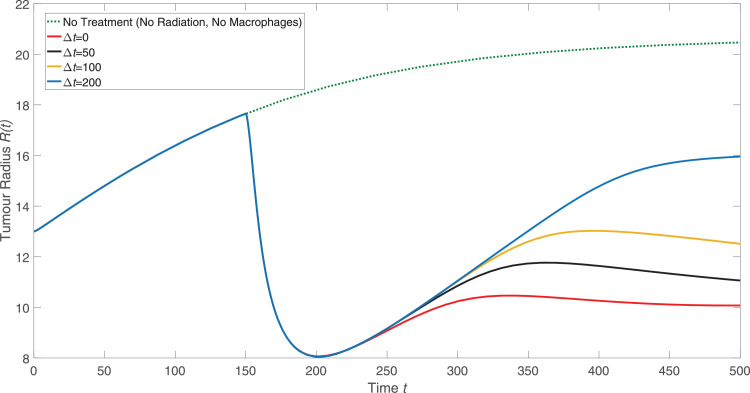
The effect of delay between radiation and introducing the engineered macrophages to the culture medium surrounding the tumour. Radiotherapy is given at t=150 as in Fig. 4. After a time delay (*Δt*) following radiotherapy, engineered macrophages were introduced into the culture medium surrounding the tumour.

For the numerical results presented in Fig. 4, there is a time delay Δt=50 between the time at which radiotherapy is administered and the time at which engineered macrophages are introduced into the extracellular material surrounding the tumour. This delay mimics the protocol used in Muthana et al. (2013). Fig. 5 shows how varying the time between radiotherapy and the macrophage-delivered virotherapy affects the tumour radius over time. When the delay reaches Δt=200, significant tumour regrowth has occurred before the virotherapy begins to take effect. If the delay is too long, the increase in chemoattractant levels caused by radiotherapy has dissipated and the tumour has sufficiently regrown that the radiotherapy no longer helps the macrophages penetrate into the tumour. The results presented in Fig. 5 suggest that the effectiveness of macrophage-delivered virotherapy monotonically decreases as the time delay between radiotherapy and macrophage therapy increases.

**Fig. 6:**
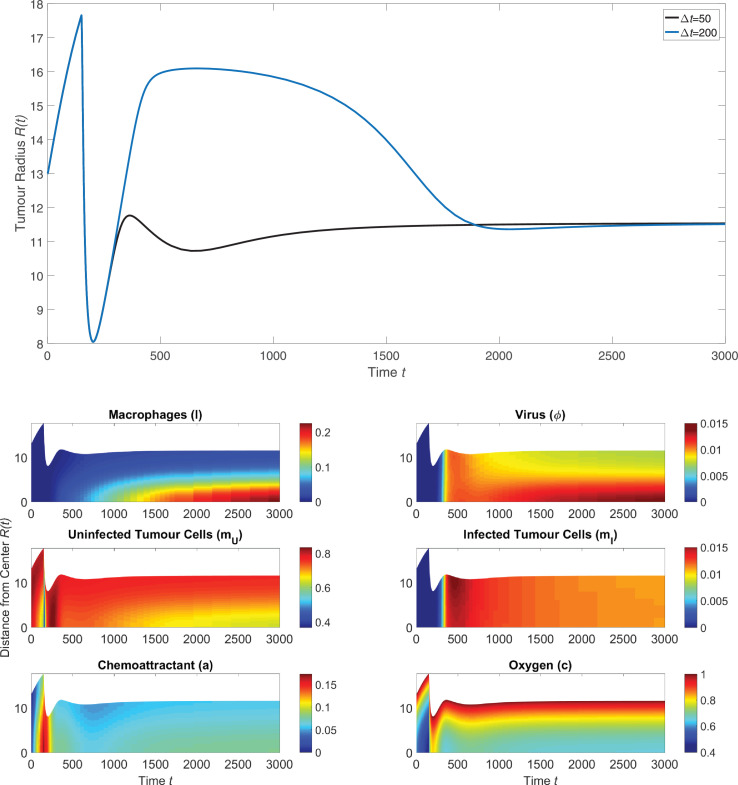
*(upper)* The long term behaviour of the tumour radius after radiotherapy is given at t=150 and engineered macrophages are introduced after a time delay *Δt* following radiation. The two curves in this panel are the same as those in Fig. 5 but are shown here over a longer timescale. *(lower)* The long-term spatial distribution of phases corresponding to a delay Δt=50 between radiotherapy and introduction of engineered macrophages.

Tumour spheroids grown in vitro will begin to shed cells into the surrounding media after a certain period of time; Leek found this time to be approximately 16 days Leek (1999). The analysis presented in this paper thus far used the restricted time domain 0 ≤ *t* ≤ 500 to reflect the limited lifespan of the tumour spheroid. Fig. 6 shows the same solution to Eqs. (43) - (50) as in Fig. 5 but over the longer timespan 0 ≤ *t* ≤ 3000. The tumour’s behaviour at later times is speculative, as the tumour is likely to begin shedding cells for *t* > 500 and this effect was not included in our model. However, these longer simulations allow for predictions of how treatment will affect a tumour in the long term.

Fig. 6(upper) shows how the tumour radius changes over a long timescale when radiotherapy is given at t=150 and the engineered macrophages are introduced after either a short delay Δt=50 or a long delay Δt=200. For the short delay, the treatment with radiotherapy followed by a macrophage-delivered oncolytic virus prevents tumour regrowth and causes the radius to tend towards a steady state. If the delay is extended to Δt=200, the tumour radius reaches the same steady state in the long term, but not before the tumour regrows almost to its pre-treatment size. Fig. 6 (lower) shows the spatial distribution of the model phases for the shorter Δt=50 delay. After the tumour cell death caused by radiotherapy, macrophages migrate into the centre of the tumour where they accumulate over time and release an oncolytic virus. The tumour cell death caused by viral infection balances tumour cell proliferation, causing the tumour size to reach a steady state.

When simulating Eqs. (43) - (50), we used one dose of radiotherapy to follow the protocol from Muthana et al. (2013). In a clinical setting, however, radiation is typically administered according to a fractionated schedule whereby multiple doses are scheduled over a period of time Fowler (1989). To investigate whether macrophage-delivered virotherapy may be more effective if it were administered between two doses of radiotherapy, we modified Eqs. (44) - (45) so that their respective net rates of production include an additional radiotherapy term. The net rates of production for Eqs. (44) - (45) become:

(51) $$\begin{array}{cc} S_{m_{I}} & =r_{\phi }\phi m_{U}-m_{I}\left[d_{m}\left(c\right)+lk\left(c\right)+k_{\phi }\right]-R\left(m_{I},t_{s}^{\prime }\right), \end{array}$$

(52) $$\begin{array}{ccc} S_{m_{U}} & = & m_{U}\left[p_{m}\left(m_{U}+m_{I},c\right)-d_{m}\left(c\right)-lk\left(c\right)-r_{\phi }\phi \right]-R\left(m_{U},t_{s}\right)\\ & & -R(m_{U},t_{s}^{\prime }). \end{array}$$

The second dose of radiotherapy starts at time $t_{s}^{\prime },$ which is assumed to be equal to, or later than, the time at which the engineered macrophages are administered. Each radiotherapy dose causes an increase in chemoattractant, so the net rate of production for Eq. (47) is also modified to include an additional radiotherapy term:

(53) $$\begin{array}{ccc} S_{a} & = & \gamma [R\left(m_{U},t_{s}\right)+R\left(m_{U},t_{s}^{\prime }\right)+R\left(m_{I},t_{s}^{\prime }\right)]\\ & & +d_{a}\left[p_{a}\left(l,m_{U}+m_{I},c\right)-a\right]. \end{array}$$

**Fig. 7:**
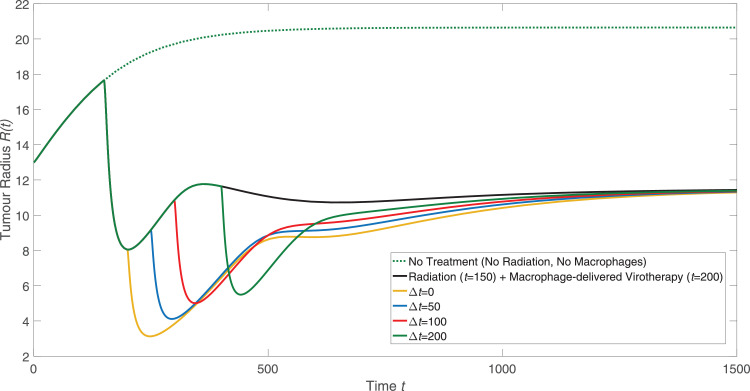
Plots showing the effect of a second dose of radiotherapy given after the engineered macrophages are introduced to the culture media. The model given by Eqs. (43) – (50), subject to the modifications in Eqs. (51) – (53), was solved using the parameters from Tables A.1 – A.2. The first dose of radiotherapy is given at t=150 and the engineered macrophages are introduced at t=200. After a time delay (*Δt*) following the introduction of engineered macrophages, a second dose of radiotherapy is given. These solutions are compared to the model solution when no radiotherapy or engineered macrophage virotherapy is given (green curve, dotted) and to the solution when only one dose of radiotherapy is given at t=150 followed by macrophage therapy at t=200 (black curve). (For interpretation of the references to colour in this figure legend, the reader is referred to the web version of this article.)

Solving Eqs. (43) - (50) subject to the changes in net rates of production given by Eqs. (51) - (53) reveals the effect of administering a second dose of radiotherapy after exposure to the engineered macrophages. The results presented in Fig. 7 show how the evolution of the tumour radius changes as we vary the time interval between applying the macrophage virotherapy and delivering the second dose of radiotherapy. Compared with a single dose of radiotherapy followed by macrophage-delivered virotherapy, the second dose of radiotherapy affects the tumour radius in the short term but does not reduce tumour size in the long term.

**Fig. 8:**
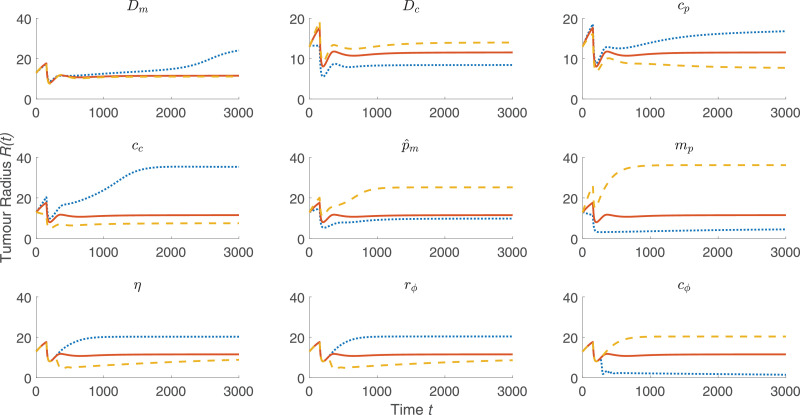
Summary of a one-at-a-time parameter sensitivity analysis of the model defined by Eqs. (43) – (50) where radiotherapy was given at t=150 and macrophage-delivered virotherapy was given at t=200. Results for the nine most sensitive parameters are shown, while the full analysis is shown in Appendix A (see Fig. A.2). Using the default parameter values specified in Appendix A, and for each model parameter, the governing equations were solved using 50% of that parameter’s default value (blue curve, dotted), the default value (red curve), and 150% of the default value (yellow curve, dashed). (For interpretation of the references to colour in this figure legend, the reader is referred to the web version of this article.)

**Fig. A2:**
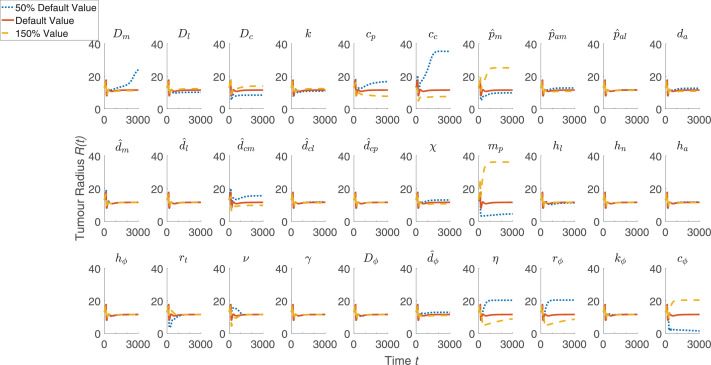
The full results of the one-at-a-time parameter sensitivity analysis to complement the summary in Fig. 8. Using the default parameter values specified in Appendix A, and for each model parameter, the governing equations were solved using 50% of that parameter’s default value (blue curve, dotted), the default value (red curve), and 150% of the default value (yellow curve, dashed) while holding all other parameters fixed at their respective default values. Solutions were numerically stable in all cases. We note that in some cases, the results are sufficiently similar that the three curves are difficult to distinguish. (For interpretation of the references to colour in this figure legend, the reader is referred to the web version of this article.)

The results of our model presented in Fig. 4, Fig. 5, Fig. 6, Fig. 7 suggest that radiotherapy is important for helping engineered macrophages penetrate into the tumour, but that the main factor in preventing tumour regrowth is the sustained tumour cell death over long timescales caused by viral infection. To investigate the importance of each parameter on the tumour size over time, we performed a one-at-a-time parameter sensitivity analysis similar to the one presented in Fig. 3. Eqs. (43) - (50) were solved using the parameters in Table A.1 - A.2 with radiotherapy given at t=150 and macrophage-delivered virotherapy given at t=200. The tumour size in the long term is most sensitive to the nine parameters that are presented in Fig. 8, which include three virotherapy parameters (*η, r_ϕ_, c_ϕ_*) but no radiotherapy parameters. The full analysis is shown in Fig. A.2.

## 5 Discussion

We have developed a new continuum model that can be used to investigate how a novel, macrophage-based virotherapy acts and how, by suitable scheduling, its combination with radiotherapy can produce synergistic treatment responses. We can identify parameter values for which the model predictions are in good qualitative and quantiative agreement with independent results from in vitro and in vivo experiments Leek (1999); Muthana et al. (2013). The model suggests that following radiotherapy, there may be finite time window during which macrophages should be introduced to produce the maximum reduction in tumour volume. Introducing the engineered macrophages within a delay of Δt=100, or approximately 5 days, after treatment with radiotherapy caused the tumour size to quickly reach a steady state as the virotherapy abolished tumour regrowth. These results qualitatively agree with the experimental results from Muthana et al. (2013). The same steady state was reached for longer delays, but over much longer timescales where the accuracy of the model is speculative. In practice, engineered macrophages should be introduced close enough after radiotherapy so that chemoattractant levels are still high and the tumour radius is still small; both factors help the macrophage-delivered virotherapy to quickly counterbalance tumour cell proliferation. It motivates further work to test whether this effect holds true in the results of in vitro and in vivo experiments. If so, this type of prediction could influence how radiotherapy and macrophage-delivered virotherapy are scheduled.

Our model suggests that radiotherapy alone is not sufficient to prevent tumour regrowth, which is consistent with the experimental observations in Muthana et al. (2013). Applying a second dose of radiotherapy after the engineered macrophages had been introduced did not effect a greater reduction in tumour size than that achieved with only one dose of radiotherapy; in both cases, the tumour size reached the same steady state by t=1500. Radiotherapy, as modelled in this paper, causes significant tumour cell death but only over a short period of time. In contrast, the way in which we have modelled oncolytic virotherapy allows for low levels of sustained tumour cell death over long timescales. While radiotherapy plays a significant role in helping macrophages deliver the oncolytic virus to the tumour, the sustained cell death caused by viral infection appears to be the critical factor in abolishing tumour regrowth in the long term.

While the long term simulations presented in this paper generate interesting predictions, our model is likely to be most accurate over shorter timescales. For the in vitro tumour spheroids considered in this paper, the tumour would begin to disintegrate by shedding cells into the surrounding media. For an in vivo tumour, the hypoxic tumour cells are likely to stimulate angiogenesis which would provide the tumour with the nutrients to fuel further growth Hubbard and Byrne (2013). In addition, the model developed in this paper applies to a tumour freely suspended in growth media. The surrounding tissue in vivo may also play a role in regulating the tumour’s growth: The tumour cells may produce proteases which degrade the surrounding tissue matrix, making it easier for the tumour to grow. These factors were excluded from our model for simplicity, but they may play an important role in how the tumour responds to treatment over long timescales.

A similar model by Jenner et al. used a system of three ODEs to model the changing levels of free virus particles, uninfected tumour cells, and infected tumour cells Jenner et al. (2018). Their model showed that adding an oncolytic virus to a growing tumour resulted in stable oscillations in the number of tumour cells rather than continued tumour growth, and that treatment with an oncolytic virus alone is not enough to completely kill the tumour. Our model further incorporates radiation and macrophage delivery, and while we do not observe oscillations in our model solution, the results from Fig. 6 also suggest that the oncolytic virus abolishes tumour regrowth but does not reduce the tumour size to zero.

The theoretical studies presented in this paper illustrate how mathematical modelling can complement treatment development: The number of possible treatment schedules and combinations is too large to investigate exhaustively with experiments, but the modelling framework presented here can pinpoint promising treatment schedules that warrant further experimental investigation. In addition to the treatment schedules investigated in this paper, the model could be used to predict the effectiveness of additional strategies such as introducing a second dose of modified macrophages at a later time.

A key contribution of this paper is illustrating how to decompose those tumour constituents or phases that contribute the most to the overall tumour volume into distinct “subphases” that share the same physical properties by using the theory of mixtures. While in this paper attention focused on the interplay between infected and uninfected tumour cells, natural extensions of the model include competition between native and engineered macrophages. Taken another step further, the model could be extended to include a cancer stem cell phase that is resistant to conventional therapy and/or a phase representing the vascular volume Hubbard and Byrne (2013). These extensions are straightforward to incorporate using the approach outlined in this paper.

## 6 Conclusions

In this paper we have derived a tumour modelling framework based on the theory of mixtures. We have used this framework to investigate the effectiveness of radiotherapy and macrophage-delivered virotherapy when administered in combination to an in vitro tumour spheroid. While additional experimental work is needed to accurately parameterise the model, our results suggest that macrophages should be introduced immediately after radiotherapy in order to produce the maximum therapeutic effect. While validating these predictions requires further experimental investigation, our model represents a useful framework for identifying promising new treatment schedules and strategies.

## Figures and Tables

**Table A1:** Summary of default values of the dimensionless parameters used to solve Eqs. (1) - (3). These parameters are used in the solutions presented in Figs. 1, 2, 3, and are complemented by the parameters in Table A.2 when the model is extended to include radiotherapy and engineered-macrophage virotherapy. Parameters marked with † are from Owen and Sherratt (1997), and parameters marked with ‡ are from Webb et al. (2007).

Parameter	Physical Description	Dimensionless Value
*D_a_*†	diffusion constant of chemoattractant	1
*D_c_*	diffusion constant of oxygen	20
*D_l_*	diffusion constant of macrophages	10
*D_m_*	diffusion constant of tumour cells	12
*k*‡	constant of proportionality for forces	1
*c_c_*	oxygen threshold	0.2
*c_p_*‡	oxygen threshold	0.6
*m_p_*	tumour cell threshold	0.65
*α*‡	switch function steepness	5
${\hat{p}}_{m}$	scales tumour cell division	0.1
${\hat{p}}_{am}$	production of chemoattractant by tumour cells	1
${\hat{p}}_{al}$	production of chemoattractant by macrophages	1
*λ_a_*‡	decay of chemoattractant	0.01
${\hat{d}}_{cl}$	consumption of oxygen by macrophages	0.5
${\hat{d}}_{cm}$	consumption of oxygen by tumour cells	0.5
${\hat{d}}_{cp}$	consumption of oxygen by tumour cell division	0.1
*χ*	strength of chemoattractive force	1100
${\hat{d}}_{l}$	death of macrophages from lack of oxygen	1
${\hat{d}}_{m}‡$	death of tumour cells from lack of oxygen	5.5
$\hat{k}$	tumour cell death by macrophage lysis	1
*h_a_*	boundary permeability to chemoattractant	0.01
*h_l_*	boundary permeability to macrophages	0.001
*h_n_*	boundary permeability to extracellular material	90
*a*_∞_‡	amount of chemoattractant in culture medium	0
*c*_∞_‡	amount of oxygen in culture medium	1
*l*_∞_	amount of macrophages in culture medium	0.2
*n*_∞_	amount of extracellular material in culture medium	0.2
*m*_0_‡	initial volume fraction of tumour cells at t=0	0.8
*R*_0_‡	initial tumour radius at t=0	13

**Table A2:** Summary of the values of the additional dimensionless parameters for the model extension that includes radiotherapy and engineered-macrophage virotherapy. These parameters are used to solve the model in Eqs. 43 - 50.

Parameter	Physical Description	Value
*ν*	strength of radiotherapy	0.2
*r_t_*	half-life of tumour cell death due to radiotherapy	0.05
*D_ϕ_*	diffusion constant of virus	1
${\hat{p}}_{\phi }$	scales release of virus by macrophages under hypoxic conditions	10
*η*	virus released by death of infected tumour cells	0.125
*r_ϕ_*	infection of uninfected tumour cells by virus	1.5
*k_ϕ_*	death of infected tumour cells due to viral infection	0.8
*c_ϕ_*	threshold for the number of tumour cells	0.6
*γ*	production of chemoattractant due to radiotherapy cell death	0.05
*ϕ*_∞_	amount of virus in culture medium	0
*h_ϕ_*	boundary permeability to virus	0.01

## Appendix A Model Parameters

Fig. A1Fig. A2Table A1Table A2
